# Data Checking of Asymmetric Catalysis Literature Using a Graph Neural Network Approach

**DOI:** 10.3390/molecules30020355

**Published:** 2025-01-16

**Authors:** Eduardo Aguilar-Bejarano, Viraj Deorukhkar, Simon Woodward

**Affiliations:** 1 GSK Carbon Neutral Laboratories for Sustainable Chemistry, Jubilee Campus, University of Nottingham, Triumph Road, Nottingham NG7 2TU, UK; eduardo.aguilar-bejarano@nottingham.ac.uk; 2 Yusuf Hamied Department of Chemistry, University of Cambridge, Lensfield Road, Cambridge CB2 1EW, UK; vd330@cam.ac.uk

**Keywords:** graph neural networks, stereochemical misassignment, database curation

## Abstract

The range of chemical databases available has dramatically increased in recent years, but the reliability and quality of their data are often negatively affected by human-error fidelity. The size of chemical databases can make manual data curation/checking of such sets time consuming; thus, automated tools to help this process are highly desirable. Herein, we propose the use of Graph Neural Networks (GNNs) to identifying potential stereochemical misassignments in the primary asymmetric catalysis literature. Our method relies on the use of an ensemble of GNN models to predict the expected stereoselectivity of exemplars for a particular asymmetric reaction. When the majority of these models do not correlate to the reported outcome, the point is labeled as a possible stereochemical misassignment. Such identified cases are few in number and more easily investigated for their cause. We demonstrate the use of this approach to spot potential literature stereochemical misassignments in the ketone products resulting from catalytic asymmetric 1,4-addition of organoboron nucleophiles to Michael acceptors in two different databases, each one using a different family of chiral ligands (bisphosphine and diene ligands). Our results demonstrate that this methodology is useful for curation of medium-sized databases, speeding this process significantly compared to complete manual curation/checking. In the datasets investigated, human expert checking was reduced to 2.2% and 3.5% of the total data exemplars.

## 1. Introduction

Within chemical informatics, the ready availability of literature experimental data, together with the surge in available data technologies has led to an explosion of chemical databases [1,2,3]. Even simple searches (Dec 2024) revealed > 100 chemically related databases. These resources are of importance as they allow the summary of experimental molecular properties, reaction outcomes, and other data into single files or web-based resources for the scientific community. Popular databases relating structures to property descriptors include PubChem (general properties) [4], ZINC (bio-medical) [5], the Delaney Solubility database (aqueous systems) [6], the tautomer database (as a function of solvent) [7], and various Quantum Chemistry derived databases (for both real and virtual molecular ensembles) [8,9,10,11,12]. Assembling and validating the data within such resources can be a hugely (human) time-intensive process.

The time committed to chemical database creation is, however, justified due to their utility in machine learning (ML) [13,14,15,16] allowing high-dimensional analysis of experimental results and finding underlying trends that may not be noticeable on initial (human) analysis of the data. ML methods have allowed some of the most challenging problems in contemporary chemistry to be solved, including protein structure prediction [17], and novel antibiotic discovery [18].

While ML models typically always benefit from larger training datasets, their performance highly depends on the quality of that primary data [19,20,21]. For chemical databases, this quality can be compromised due to human errors, both in the original literature and upon data entry, including the following: (i) the molecule’s structure has not been transcribed correctly into the database; (ii) mismatching of the database molecule to its genuine measured property (i.e., through swapping, deletion or miss-entry of primary data) [22]. These errors are potentially harmful for ML model training purposes, and ideally, they should be corrected before model development to avoid invalid molecular representation–target variable relationships developing.

For chemical reaction database auto-curation, the present efforts have focused on the use of algorithms and templates to identify potential structural errors within the reactants in the database [23,24,25]. Gimadiev et al. described an algorithm to correct entered chemical structures and transformations [23]. To do that, they described a protocol that allowed standardization of chemical reaction notation, including the following: (1) standardizing functional groups using standard representations (for example, S^+^-O^−^ bonds are rewritten as S=O), (2) standardization of aromaticity by writing smiles strings in Kekulé form (for example, for benzene, C1=CC=CC=C1), and rewriting the molecule as aromatic again (c1ccccc1 for benzene), (3) elimination of reactions where a participant molecule has any atom with a violated valence (for example, carbon atoms with more than four bonds), (4) rewriting the molecules in terms of the major tautomer (if they exhibit tautomerism). In a related study, Chen and Li developed an algorithm that automatically identifies meaningful reaction transformation rules within the dataset of interest. Such rules are encoded using SMARTS [24], which then serves as a template to identify problems in the entries of the dataset, such as missing atoms, reactants, and incorrect atom mappings [24]. In a study relevant to our own, Toniato and co-workers offer a proof that it is possible to identify outliers in chemical reaction outcomes by identifying those data points where the model struggles the most to learn [25]. This is similar to the case where large language grammar errors can be identified through differential learning rates. Although effective, none of the above studies discusses the more basic problem of curating (human) mislabeled data in such reaction datasets.

We can identify only two previous attempts to use Graph Neural Networks (GNNs) to identify mislabeled data in chemical databases [22,26]. Garrison et al., when modeling the features of ca. 86 thousand calculated coordination compounds [27], used different GNN architectures to identify a subset of complexes whose predicted properties were poor in all models. By manual inspection, they found that these shared an energy underestimation caused by too low a level of DFT calculation within the original tmQM calculated database. The GNN identified curation errors which required improved basis set and level calculations to be made (from TPSSh-D3BJ/def2-SVP to ωB97M-V/def2-SVPD level) [26]. Similarly, Ulrich and co-workers trained different ML models (including GNNs), using a lipophilicity (logP) database. They identified 63 compounds, where at least four of the ML methods predicted logP values significantly different from the experimental values. Manual inspection revealed these datapoints were incorrectly labeled in the primary data [22]. Given this context, we hypothesize that GNNs might be useful in identifying possible stereochemical misassignments in the primary asymmetric catalysis literature.

Herein, we use Graph Neural Networks to identify the potential stereochemical misassignments in in exemplary asymmetric catalysis literature [28]. Our approach consists of the training of an ensemble of GNN models using a nested cross-validation approach and identifying such datapoints where the majority of models predicts stereochemical assignment with high error (compared to the literature apparent “ground truth”). Once identified, manual inspection of such exemplars (a manageable minority set) can be carried out and the origin of the discrepancy can often be discovered, allowing its correction or removal from the dataset as desired. This approach assumes that misassignments are always associated with atypical (outlier) behavior and that the GNN effectively models the bulk of the data successfully. We evaluate this procedure using two rhodium-catalyzed asymmetric 1,4-addition databases. Each comprised a different family of ligands, either chiral dienes (688 exemplars reactions) or chiral bisphosphines (644 exemplars). We demonstrate that our methodology allows accelerated data correction/curation, while the outliers identified can provide interesting information in their own right.

## 2. Results and Discussion

We used two databases constructed from a literature review on “Enantioselective, Rhodium-Catalyzed 1,4-Addition of Organoboron Reagents to Electron Deficient Alkenes” (see Section 4.1) [28]. Using a nested cross-validation approach, we trained an ensemble of models to predict various test fold enatioselectivities (see Section 4.2, 4.3). We used the HCat-GNet GNN [29,30] architecture as it has demonstrated good performance in a range of asymmetric reactions and it is computationally inexpensive to implement. We created graph representations of the reactions using HCat-GNet (see Section 4.4) and then trained the models to predict the “%top” as the stereochemical outcome variable (see Section 4.5). We choose %top as it is independent of the substrate and reagent structures in a way that the Chan–Ingold–Prelog classical stereochemistry descriptors *R* and *S* are not [31]. For each training–testing process, we identified the outliers (typically < 5% of the data exemplars) by a suitable criterion (see Section 4.6), and counted how many times a single point was considered an outlier. If a point was considered an outlier five or more times (against nine models), then the point is considered a potential stereochemical misassignment and investigated manually (in both the review [28] and original reference). Below, we present the results attained for each database in separate sections. For each dataset section, we separate the results by (1) model performance and (2) outlier identification.

### 2.1. Diene Dataset

#### 2.1.1. Results of the Test Points in the Nested Cross Validation

**Figure 1:**
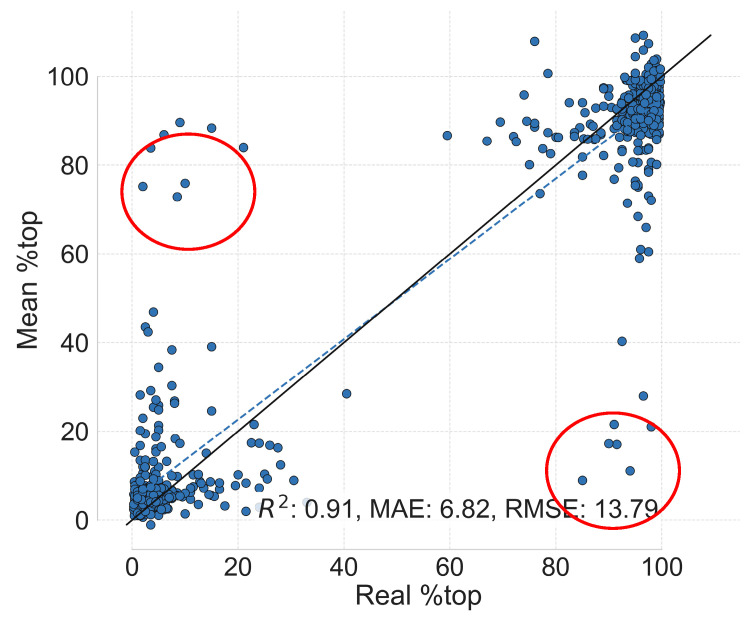
Parity plot of “%top” for all test data points from the nested cross-validation data splitting strategy applied to the diene dataset. Each data point represents one reaction, and we summarize all GNN predictions of a single reaction by calculating the mean (see Section 4). The outliers circled represent, at most, 3% of the dataset. The solid and dashed plots are the theoretical and fitted lines of equality respectively.

We trained the GNN models on the diene database (688 exemplars, with 10 fold split) following the procedure indicated in the Materials and Methods section. Our outlier identification algorithm relies on a voting system, so we summarized the prediction of all the different models by using the mean of the predictions. Figure 1 shows the parity plot attained from this process.

In Figure 1, it is clear the model detects the correlation between the reaction graph representations and the “%top” outcome for the majority of points. The metrics obtained are comparable with other methods that model the enantiomeric ratio of related reactions [31,32,33,34], indicating that HCat-GNet is reliable in this predictive task. The circled data points indicate that the model struggles to predict the (nominally ground truth) “%top” variable for a small minority of the points. We manually inspected those points and found there was (1) clear (literature) misassignment, (2) that circumstantial evidence points to a potential stereochemical misassignment, or (3) that some other unusual factor being at play. We could identify no clear false positives selected by our algorithm.

#### 2.1.2. Outlier Analysis

We list below the reactions that were found to be potential stereochemical misassignments by HCat-GNet. The reaction index ID (i.e., that for the reaction in the database given in the SI to this paper), as well as the quantity of times that point was considered as an outlier (out of nine data folds), are given in Table 1.

From the 688 reactions in the original diene database, we were able to reduce the manual data curation space to only 15 reactions (around 2% of the total). This makes the data curation significantly faster and manageable to do by manual inspection. For example, we found that in reaction IDs 229 and 572, the absolute stereochemistry of the product was not actually explicitly assigned in the original primary literature [28,36]. This means that the error arose during database building, when a human curator presumed a stereochemical outcome that was not present. The fact that these reactions were mis-predicted nine times by the GNN means that is very likely that the reaction’s true outcome is the opposite of the original database entry. Thus, it is possible to use HCat-GNet as a stereochemical assignment tool for cases where explicit experimental stereochemical correlations are not present, using a “wisdom of crowds” approach.

**Figure 2:**
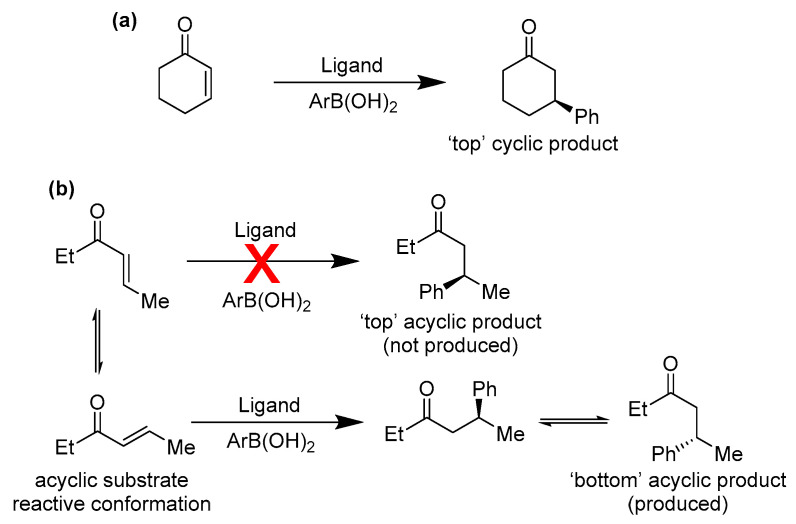
Reactions where the stereoisomer of the product is inversed in terms of the top and bottom stereoisomers for cyclic and acyclic substrates. (**a**) Shows the reaction for a fixed *cis* conformer of a cyclic substrate, and (**b**) shows the reaction for an acyclic substrate that can react in both *s-cis* and *s-trans* conformations.

Human expertise is still needed in some adjudications. For example, reaction IDs 320 and 663, by manual inspection, also reveal an apparent stereochemical inversion for experimental observation vs. predicted values [40]. For these reactions the GNN has only two substrates to learn from: cyclohex-2-en-1-one (cyclic, Figure 2a) and (*E*)-hex-4-en-3-one (acyclic, Figure 2b). Stereochemical reversal between cyclic and acyclic is a known phenomenon, especially for acyclic substituents weighted strongly towards *s-cis* vs. the more common *s-trans* conformations (Figure 2). Is likely that our GNN did not learn such a rule (due to the limited available data) and could only deduce that the stereochemical outcome is ligand dominated (regardless of the nature of the substrate). Therefore, these points are not considered stereochemical misassignments.

**Figure 3:**
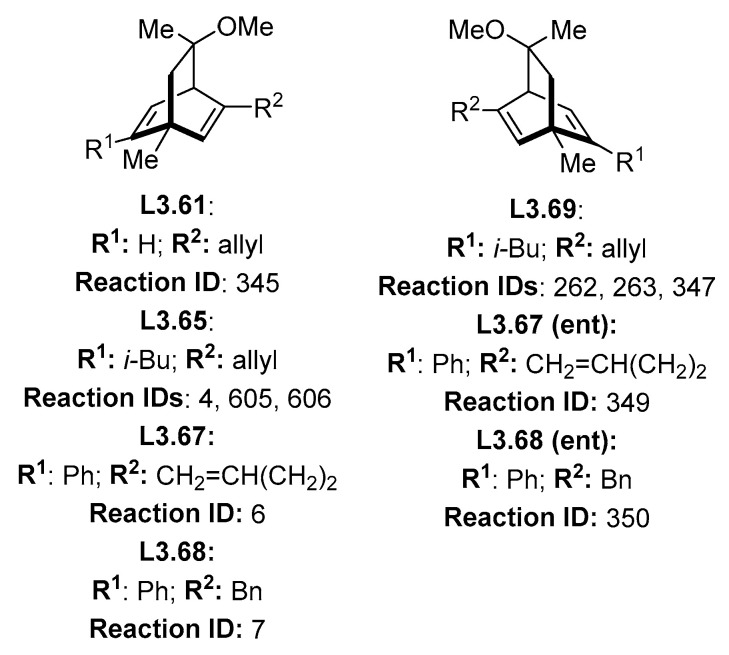
Dolefin-type ligands used in reactions that the HCat-GNet model found potential stereochemical conflicts to those assigned in the literature.

In the case of reaction IDs 4, 6, 7, 262, 263, 345, 347, 349, 350, 605, and 606 (nomenclature of our database in Supplementary Information), all of these use catalysts share a common core structure (Figure 3). Very interestingly, prediction of the enantioselectivities provided by these ligands was also problematic in previous ML studies [31]. As identified in the Lam review [28], it is notable that all of these results come from just three papers, two of which are from the same research laboratory [35,37,39].

**Figure 4:**
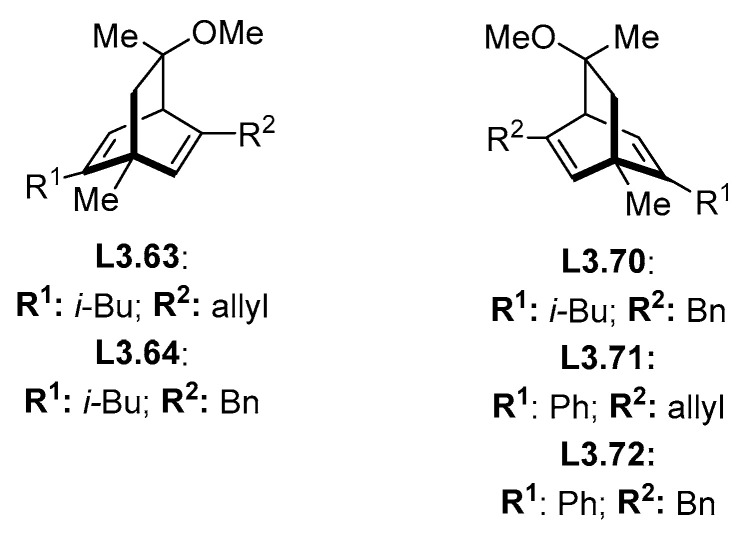
Dolefin-type ligands used in reactions that the model could predict accurately the stereoisomer produced.

While it could be possible that ligands in Figure 3 present completely different behavior compared to all other ligands (as judged by HCat-GNet), such behavior in globally closely related diene ligands would be unusual. Therefore, we checked the database to identify other reports of reactions using the same class of ligands that describe (re)analysis of the reported stereochemical outcome. Unfortunately, none existed for the explicit ligands of Figure 3. However, we could identify other dolefin-type ligands used in reactions in our database (Figure 4) with correlated product stereochemistry. These dolefin ligand structures which did not suffer miscorrelation and were not considered outliers are shown in Figure 4.

In all the reactions that used dolefin ligands, we noticed that all the reported reactions for **L3.63** were carried out within one group [35]. They stated that ligands showing the stereochemistry of **L3.63** (Figure 4) generate only “top” isomers in our nomenclature (i.e., formation of the *S* enantiomer for the addition of PhB(OH)_2_ to cyclohexanone). In the case of **L3.64**, two different studies from Carnell and co-workers [41,42] stated that the identical ligand gave the “bottom” isomer for cyclic substrates. For **L3.70**, in a total of 42 reactions from two different papers from Carreira and co-workers [37,43], it is stated that the produced enantiomer is “top” for acyclic substrates. For **L3.71**, three reactions from the same sources [37,43] correlate such a ligand configuration to the “top” product for acyclic substrates [37,43]. Lastly, for **L3.72,** only one reaction reports the correlation between the ligand configuration: this is for a “top” product from an acyclic substrate. From all the papers of Carreira, their general rule is dolefin ligands with the stereoconfiguration of **L3.61**, **L3.63**, **L3.64**, **L3.65**, and **L3.67** (called here configuration-2) generating the “top” isomer for cyclic substrates and “bottom” for acyclic substrates. Enantiomeric ligands **L3.69**, **L3.70**, **L3.71**, and **L3.72** (called here configuration-1) generate the opposite selectivity (“bottom” for cyclic substrates) [35,37,43]. This selectivity is opposite to that reported by Carnell and co-workers [41,42]. By checking general trends in the database, we noticed that the general consensus in the relationship between ligand planar chirality and stereochemical outcome is that ligands with configuration-2 generate “bottom” products when reacting with cyclic substrates and “bottom” when reacting with acyclic acceptors. Such latter assignments are also in accord with Hayashi’s empirical selectivity model [44].

We investigated this issue further using a natural product synthesis of hybridalactone (of known stereochemistry) by Fürstner and co-workers [39]. For the initial step of the synthesis, they report using **L3.69** to generate the “top” product of the addition to the 2(*5H*)-furanone substrate (ID 263 in our database). This addition follows the rule of the configuration-1 ligand’s generating “top” selectivity for cyclic substrates. As this paper reports a known natural product is very likely, its stereochemical assignment is correct, implying that the other reports of dolefin ligands where the rule is inverted in the literature are very likely to be stereochemical misassignments. The fact that HCat-GNet flags this reaction as a possible misassignment is due to the quantity of dolefin ligands inverting the rule, biasing the models to think this class of ligands invert the stereochemical rule.

In summary, for the problematic assignments of Table 1: two of the 15 (IDs 229 and 572) are confirmed as stereochemical inversion errors, 11 (IDs 4, 6, 7, 262, 263, 345, 347, 349, 350, 605, 606) are very likely (but not confirmable without further experimental work) inversion errors, and for two (IDs 320, 663), insufficient training data for accurate assessment existed. While all the outliers identified by HCat-GNet were analyzed manually, this is reduced to a manageable level of human intervention. As demonstrated in our discussion, not every outlier for the model means that the datapoint (reaction) was wrongly reported.

### 2.2. The Bisphosphine Database

#### 2.2.1. Results of the Test Points in the Nested Cross Validation

**Figure 5:**
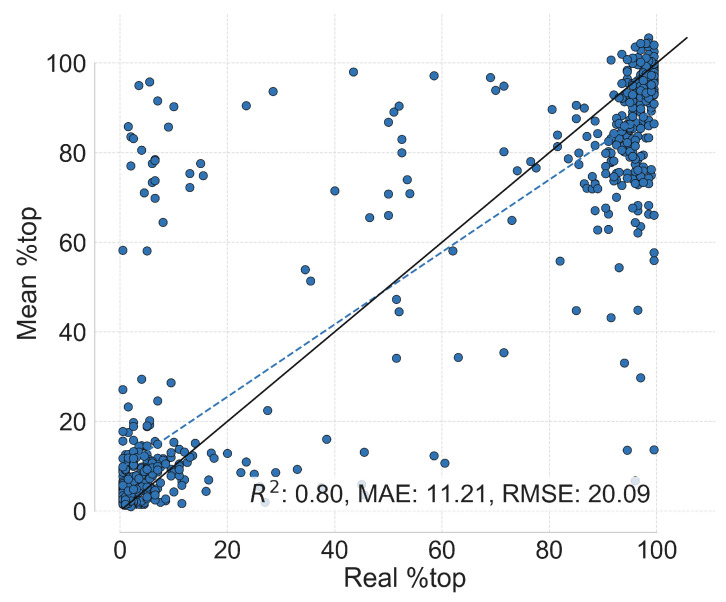
Parity plot of “%top” for all test datapoints from the nested cross-validation data splitting strategy applied to the bisphosphine dataset. Each data point represents one reaction, and we summarize all GNN predictions of a single reaction by calculating the mean (see Section 4). The solid and dashed plots are the theoretical and fitted lines of equality respectively.

Using the same approach (see Section 2.1), we trained our HCat-GNet models on a bisphosphine dataset attained from all the data on this ligand class present in the Lam review [28] and used this to identify outliers. Figure 5 summarizes the results obtained.

Figure 5 demonstrates that HCat-GNet does not model the bisphosphine dataset as well as it did for the diene database. When creating this new database, we were fastidious in transcribing and checking the data to avoid this as a source of additional outliers. Thus, the outliers of Figure 5 can only occur for two reasons: (1) the catalyst–substrate interactions are so subtly complex that they are no not modeled effectively by the GNN, or (2) there are misassignments in the primary literature describing the stereochemistry of the products. We discuss disentangling these for the various outliers in the following section.

#### 2.2.2. Outlier Analysis and Database Curation

As in the case of the diene dataset, we summarize the outliers that were labeled as possible stereochemistry misassignments in Table 2.

**Figure 6:**
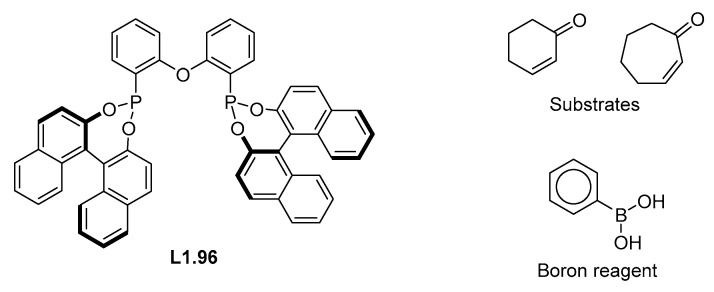
Under represented ligand **L1.96** and the acceptors and the boron reagent used with these.

In the case of the reactions that used ligand **L1.96** (IDs 40 and 196), we found that the model struggled to predict the correct face of nucleophile addition. As the substrate and boron reagent used in both reactions are well represented in the database (structures within Figure 6), is likely that error results from the model inaccurately describing the stereoselectivity engendered by the structure of this ligand (Figure 6). This is most likely due to the low representation of samples using this unusual catalyst class (only two such reactions are in the dataset).

**Figure 7:**
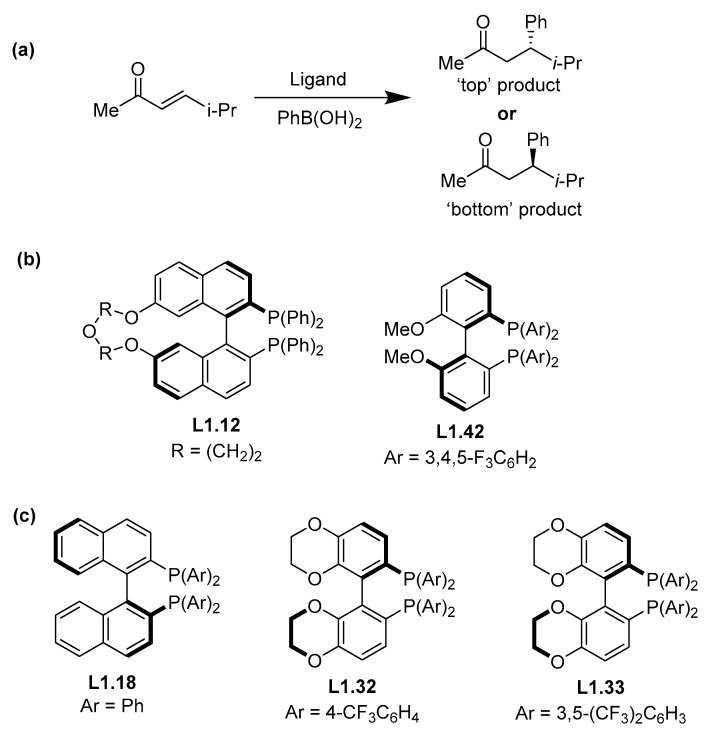
Enantioselective transformation of (*E*)-5-methylhex-3-en-2-one into 5-methyl-4-phenylhexan-2-one by different catalysts. (**a**) Shows the general reaction, (**b**) the ligands that report the “bottom” addition product, and (**c**) ligands that report “top” addition.

In the case of reaction IDs 185 and 191, both of these share the same substrate (*E*-5-methylhex-3-en-2-one) and boron reagent (PhB(OH)_2_); see Figure 7a. This substrate is particularly problematic within our database. As alluded to before (Figure 2), acyclic acceptors can react in an *s-cis* conformation so that the product is the inverse stereoisomer to that from a cyclic acceptor. Unfortunately, some researchers suggest that conformational exchange does not invert product chirality [47,58,59,60,61,62], while others say it does [45,46,63]. To demonstrate this problem, highlighting the discrepancies in the literature, we took a subset of reactions with the same substrate and boron reagent (reaction IDs 181, 184, 188, 189, and 190 in our dataset) with varying catalyst ligands (Figure 7).

Typically, ligands with *S* axial chirality (equivalent to configuration 1) (e.g., **L1.18**) generate “top” products with cyclic acceptors while ligands with *R* axial chirality (configuration 2) (e.g., **L1.12**, **L1.42**, **L1.32**, and **L1.33**) generate the “bottom” Michael addition products. Thus, the facial enantioselectivity of the addition process is mainly driven by the axial chirality of the ligand. Given this, it would be expected that if two ligands having the same axial chirality and used with the same substrate and boron reagent pair under near identical conditions, would generate the same product stereochemistry, e.g., the product generated by **L1.18** (reaction ID 181) should be the enantiomer of the product generated by **L1.12**, **L1.42**, **L1.32,** and **L1.33**. Unfortunately, the literature results for **L1.12** and **L1.42** indicate the generation of the “bottom” addition product for this substrate [59,61], while the rest of ligands of Figure 7 generate the “top” product [58,63]. It could be the case that the difference in the isomer produced are due to differences in the ligand structure; however, this is unlikely, as the main driver of the enantioselectivity is the axial chirality, which is also supported by the simple quadrant model by Hayashi [44]. From our analysis, we suspect that there are misassignments in the stereochemistry of the products from this subset of reactions, but explicit re-investigation would be required. It is possible in reaction IDs 185 and 191 that this might be caused by a poorer fit of this particular substrate due to misassignments in reaction IDs 181, 184, 188, 189, and 190.

For the reaction ID sets (222, 223, 228, 229, 230, and 233) vs. (287, 289, 328, 346, 351, 403, and 425), which also use acyclic acceptors, we propose that a similar situation has occurred—although here we can only flag the data for further investigation and not provide a clear dissection on the data alone.

For reaction IDs 512, 536, and 538 that use ligand **L1.18**, there are also data anomalies. In the case of reaction IDs 512 and 536, while the substrates were not cyclic, the reported chirality outcome is “bottom” (for an *S* axial chiral bisphosphine). For reaction ID 536, although the substrate is cyclic, the reported product is “top” (for an *S* axial chiral bisphosphine), while the rest of cyclic substrates are reported to be transformed into a “bottom” product by this ligand (**L1.18**). This latter reaction is very likely to be a case of stereochemical misassignment. Lastly, for reactions using **L1.66**, no anomalies were apparent to us.

In summary, for the problematic assignments of Table 2: two of the 22 (IDs 536 and 5538) are likely stereochemical inversion errors, 15 (IDs 185, 191, and 222–425) appear to be due to a mixture of misassignment and subtle ligand effects (not easily analyzable without further studies), and for five (IDs 40, 196, 512, 597, 605) insufficient training data for accurate assessment existed.

## 3. Conclusions

In helping us prepare datasets for ML investigations of asymmetric catalysis, we wanted a method to help us spot possible (human) stereochemical misassignments of products within the asymmetric catalysis literature, rather than the tedious manual re-checking of all data entries against literature sources. Our present method consists of the training of an ensemble of GNN models that each use different training and validation sets. In changing these sets, we effectively are trying to gather differing chemical information predictions for each model. In our case, all of the models are used to predict the enantioselectivity of the test set. Our criterion for assigning a potential stereochemical misassignment is when the majority of models (trained on the remaining data) are at odds with a specific reaction. While this could simply be a failure of the GNN model, in our experience, it more frequently expose interesting issues in the creation and curation of the primary literature data.

Upon testing our primary diene dataset (688 exemplars), we could identify a very minor (~2.2% of the data) subset of ligands that that our GNN model could not predict the major isomer product well. While it is potentially possible that these ligands cannot be modeled by HCat-GNet, they are all very similar in core structure to the rest of the database entries. One simple alternative is that these outliers are misassignments in the primary literature. As the majority of the inconsistencies can be traced to just two studies, the possibility of an accidental (but internally consistent within the affected laboratories) stereochemical inversion is possible.

The bisphosphorus dataset is more challenging to model with simple GNN systems. We were able to identify the reasons for some of the outliers, especially reactions where the acceptor substrate was acyclic. Such substrates presented a problem for the GNN as substrate conformation weighting significantly affects the derived enantioselectivity and the primary literature itself is contradictory in this area. This introduces significant noise into the dataset, limiting the GNN’s ability to learn good correlations between structure of the ligand substrate structures and product enantioselectivity. However, thanks to the GNN and our methodology, it was possible to quickly identify this issue from the primary literature, which is also a fruitful area for further investigations.

It is always challenging to identify misassignments in stereochemistry without retrospective duplication of reactions and recourse to crystallographic studies that are hugely time intensive. The approach here is, however, effective in quickly identifying sub-set anomalous cases rapidly. This allows human investigation to focus on these rather than tedious re-checking of the whole dataset. Additionally, our study reinforces the importance of reporting stereochemical results in asymmetric catalysis. As can be seen in the bisphosphine dataset here, a few misassignments can have a disproportionate effect on the quality of ML predictions.

In principle, GNN data fidelity checking might be applied to a wide range of chemical datasets (if suitable graph representations can be attained). For example, applying this approach to heterogeneous catalysis data fidelity checking should be possible provided efficient (automated) translation of the primary data [e.g., large language model based systems such as www.digcat.org (accessed on 13 January 2025) to graph representations is possible.

## 4. Materials and Methods

### 4.1. Databases

Our primary data were from a review on “Enantioselective, Rhodium-Catalyzed 1,4-Addition of Organoboron Reagents to Electron Deficient Alkenes” by Burns and Lam [28]. We built two different databases based on the nature of the ligands used in those reactions. The first dataset consists of reactions using bisphosphine-type ligands (chelate coordination of the ligand to the rhodium center). This dataset consisted of 644 reactions, using 128 different substrates, 109 different boron reagents, and 62 different ligands. The second dataset consists of reactions using diene ligands (coordination through the two π-bonds to rhodium). We reported and used this latter dataset in previous ML studies [31]. We used an augmented version of that dataset, which was augmented by creating virtual reactions consisting of the enantiomer of the ligand used in the original study. This augmented database comprised 86 different substrates, 71 organoboron reagents, and 82 ligands applied to a total of 343 real-world reactions (688 in the augmented version). Both databases are available as Supplementary Information Files to this publication.

### 4.2. Data Splitting Strategy

We applied a nested cross-validation approach to conduct the experiments. This approach is useful for the data curation procedure because of two main reasons: (1) it allows use of each data point of the database as the test set multiple times, and (2) it allows training of ensembles of models enabling multiple predictions of each data point. This splitting strategy consists in dividing the dataset into *k* folds and utilizing each fold as a test set, but also in each test set, each of the remaining *k*-1 folds can be used as a validation set once, while the remaining *k*-2 folds will be used as the training set. This way, for each test fold, a total of *k*-1 training different processes can be performed, each with different training and validation sets. We created 10 folds, so there were 10 test sets, and within each test set, there were 9 different training processes used for validation and training.

### 4.3. GNN Model

We used HCat-GNet, with its original architecture and training mode [29,30]. The architecture of this model converts a molecular graph input into a predicted selectivity outcome by the following steps:

1. The node features are taken and are expanded to a final length of 64 by a graph convolutional operator with leaky ReLU activation function, R^nodes×24^ → R^nodes×64^.
2. The graph convolution operator updates all the node states in parallel to update the node features to graph-aware features once with leaky ReLU activation function, R^nodes×64^ → R^nodes×64^.
3. Mean and max pooling is applied elementwise to all the node feature vectors to obtain a graph-level feature vector, R^nodes×64^ → R^1×128^.
4. A fully connected layer with leaky ReLU activation function takes the graph-level vector and maps it to half of its length, R^1×128^ → R^1×64^.
5. A last fully connected layer transforms the feature vector into a scalar number, this being the prediction of the model, R^1×64^ → R^1^.

For training, an Adam optimizer was used, using the root mean squared error as the loss function. In each epoch, the loss of the validation set was supervised for two reasons: learning rate adjustment and to allow early stopping. In cases where there was no improvement in the validation loss for 5 epochs, the learning rate was multiplied by a factor of ×0.7. At the beginning of the training, the learning rate was set to 0.01 and the minimum learning rate allowed was set to 10^−8^. In cases where there was no improvement in the validation set for 30 epochs, then the training was stopped. For the learning process, the training set was delivered to the model in batches of 40 graphs, the loss and gradients were calculated for each batch, and weights and biases were updated after each batch. In each epoch, all the structures of the training set were also delivered into the batches, and an epoch was finalized once all batches had gone through their backpropagation processes.

### 4.4. Graph Representation

In generating molecular representations, the algorithm iterates over all the atoms within a component molecule. For each atom (node), the algorithm retains information on it and another atom sharing a bond with it, its atomic properties, including its identity, degree (number of non-hydrogen atoms are attached to it), hybridization, whether or not it is part of an aromatic system, whether or not it is part of a ring, and the absolute configuration of the stereocenter at that atom (either *R*, *S* or none). Component connectivity information is transcribed into an adjacency list, while the atomic property information is retained in a node feature matrix, one-hot encoded. The adjacency list along with the node feature matrix is the graph representation of the molecule (see Supplementary Information Figure S1). This pre-processing is conducted automatically for all molecule components within the reaction dataset.

### 4.5. Target Variable

**Figure 8:**
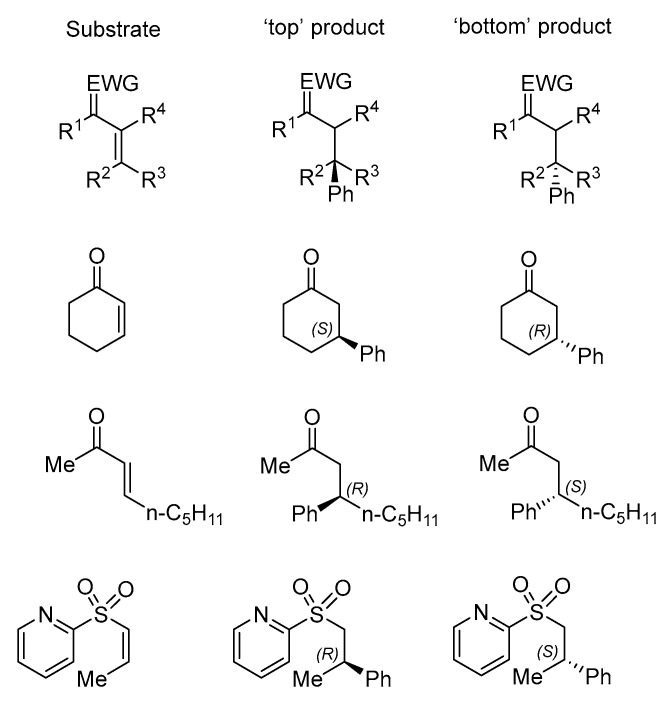
Scheme used to label a product as a “top” or “bottom” for representative substrates within the databases. This assignment allows for both enantiomeric ratio (expressed in terms of the top product) and major product (“%top” > 50 implies the “top” isomer is the major product) to be represented.

As we aim to curate stereochemical misassignments in the asymmetric catalysis literature, we selected the target variable “%top” [31]. This variable consists of the percentage of the addition of the nucleophile to the “top” face of the substrate, as defined by the face of the substrate seen when placing the electron-withdrawing group (EWG) on the top left corner of the alkene, as shown in Figure 8.

### 4.6. Outlier Threshold Criteria

For each training process, we defined that a test point was an outlier if the error of the predicted value of “%top” was three times greater than three times the standard deviation of the error values of all predictions in the test set. We selected this criterion rather than a simple error threshold (just a value deviation in “%top” for example), as the standard deviation considers the error around all the test sets. This way, if for any reason the prediction accuracy is poor in all the reactions, none of them is considered as an outlier. Also, this method drives attention to those points where the error is higher in comparison to the rest, thus reducing the quantity of total outliers and focusing the attention on those more-likely errors. The outliers of each run were identified and counted. We investigated further if an outlier was considered an outlier in five or more of nine models, effectively using the ensemble of models as a votive system where if the majority says a point is an outlier, and then it is considered as an outlier.

## Figures and Tables

**Table 1:** Reaction index and number of times it was considered as outlier when applying the outlier identification methodology on the diene dataset. We present those data points where they were outliers 5 or more times.

Reaction Index (ID) ^a^	Ligand Used ^b^	Times as Outlier	Reference ^c^
4	**L3.65**	9	(246) [35]
6	**L3.67**	8	(246) [35]
7	**L3.68**	9	(246) [35]
229	**L3.47**	9	(51) [36]
262	**L3.69**	8	(145) [37]
263	**L3.69**	8	(197, 306) [38,39]
320	**L3.81**	9	(270) [40]
345	**L3.61**	7	(246) [35]
347	**L3.65**	9	(246) [35]
349	**L3.67**	9	(246) [35]
350	**L3.68**	9	(246) [35]
572	**L3.47**	9	(51) [36]
605	**L3.69**	8	(145) [37]
606	**L3.69**	9	(306) [38]
663	**L3.81**	8	(270) [40]

^a^ The reaction index (ID) corresponds to that given in our dataset, which is available as Supporting Information of this paper. ^b^ The ligand identification code used is the same as that in the primary Lam review [28]. ^c^ Numbers in parentheses are the reference numbers in the original Lam review [25]; the superscripted numbers are the references cited in this paper.

**Table 2:** Reaction index (ID) and number of times it is reported an outlier (out of 9) in bisphosphine dataset GNN analysis. We present those datapoints where they were outliers 5 or more times.

Reaction Index (ID) ^a^	Ligand Used ^b^	Times as Outlier	Reference ^c^
40	**L1.96**	8	(242) [45]
185	**L1.68**	8	(281) [46]
191	**L1.28**	7	(78) [47]
196	**L1.96**	7	(242) [45]
222	**L1.28**	6	(78) [47]
223	**L1.34**	5	(78) [47]
228	**L1.22**	9	(278) [48]
229	**L1.24**	8	(278) [48]
230	**L1.45**	5	(278) [48]
233	**L1.1**	9	(57) [49]
287	**L1.18**	8	(66) [50]
289	**L1.18**	5	(66) [50]
328	**L1.18**	9	(257) [51]
346	**L1.18**	8	(190) [52]
351	**L1.18**	9	(190) [52]
403	**L1.66**	8	(35) [53]
425	**L1.66**	9	(35) [53]
512	**L1.18**	9	(155) [54]
536	**L1.18**	9	(66) [50]
538	**L1.18**	7	(198) [55]
597	**L1.66**	5	(36) [56]
605	**L1.66**	8	(310) [57]

^a^ The reaction index (ID) corresponds to that given in our dataset, which is available as Supporting Information of this paper. ^b^ The ligand identification code used is the same as that in the primary Lam review [28]. ^c^ Numbers in parentheses are the reference numbers in the original Lam review [25]; the superscripted numbers are the references cited in this paper.

## Data Availability

All the code used to carry out these experiments is available free of charge on Github: https://github.com/EdAguilarB/GNN_DataCuration (accessed 13 January 2025).

## Supplementary Materials

The following supporting information can be downloaded at: https://github.com/EdAguilarB/GNN_DataCuration (accessed 13 January 2025). Dataset S1: Diene dataset (rhcaa); Dataset S2: Bisphosphine dataset (biAryl).
